# Financial-related discrimination and socioeconomic inequalities in psychological well-being related measures: a longitudinal study

**DOI:** 10.1186/s12889-024-18417-w

**Published:** 2024-04-11

**Authors:** Lucy Bridson, Eric Robinson, I Gusti Ngurah Edi Putra

**Affiliations:** Department of Psychology, Institute of Population Health, University of Liverpool, Bedford Street South, L69 7ZA Liverpool, UK

**Keywords:** Financial-related discrimination, Stigma, Mental health, Socioeconomic status, Health inequalities

## Abstract

**Background:**

This study examined the prospective association between financial-related discrimination and psychological well-being related measures and assessed the role of financial-related discrimination in explaining socioeconomic inequalities in psychological well-being related measures.

**Methods:**

Data of UK older adults (≥ 50 years) from the English Longitudinal Study of Ageing were used (baseline: Wave 5, 2010/2011; *n* = 8,988). The baseline total non-pension wealth (in tertiles: poorest, middle, richest) was used as a socioeconomic status (SES) measure. Financial-related discrimination at baseline was defined as participants who reported they had been discriminated against due to their financial status. Five psychological well-being related measures (depressive symptoms, enjoyment of life, eudemonic well-being, life satisfaction and loneliness) were examined prospectively across different follow-up periods (Waves 6, 2012/2013, 2-year follow-up; and 7, 2014/2015, 4-year follow-up). Regression models assessed associations between wealth, financial-related discrimination, and follow-up psychological measures, controlling for sociodemographic covariates and baseline psychological measures (for longitudinal associations). Mediation analysis informed how much (%) the association between wealth and psychological well-being related measures was explained by financial-related discrimination.

**Results:**

Participants from the poorest, but not middle, (vs. richest) wealth groups were more likely to experience financial-related discrimination (OR = 1.97; 95%CI = 1.49, 2.59). The poorest (vs. richest) wealth was also longitudinally associated with increased depressive symptoms and decreased enjoyment of life, eudemonic well-being and life satisfaction in both 2-year and 4-year follow-ups, and increased loneliness at 4-year follow-up. Experiencing financial-related discrimination was longitudinally associated with greater depressive symptoms and loneliness, and lower enjoyment of life across follow-up periods. Findings from mediation analysis indicated that financial-related discrimination explained 3–8% of the longitudinal associations between wealth (poorest vs. richest) and psychological well-being related measures.

**Conclusions:**

Financial-related discrimination is associated with worse psychological well-being and explains a small proportion of socioeconomic inequalities in psychological well-being.

**Supplementary Information:**

The online version contains supplementary material available at 10.1186/s12889-024-18417-w.

## Introduction

Globally, the prevalence of mental health problems (e.g., depressive, anxiety disorders) increased by 48% between 1990 and 2019 [1], and this has become a major public health problem [2]. Furthermore, socioeconomic inequalities in mental health, including psychological factors related to well-being, have widened. Across multiple British population surveys, the prevalence of psychological distress in the most deprived areas was estimated to be double that of the least deprived areas [3]. In line with this, a meta-analysis indicated significant socioeconomic inequalities in depression, whereby individuals from a range of lower socioeconomic status (SES) indicators (e.g., education, income), had higher odds of being depressed than their higher SES counterparts [4]. These SES-based inequalities are consistent across a range of psychological well-being related measures, including enjoyment of life [5], life satisfaction [6], and loneliness [7]. Therefore, further understanding socioeconomic inequalities in psychological well-being is vital to inform targeted public health interventions aimed at reducing these inequalities.

A previous study used data from the UK Whitehall II Study and found that social support, material disadvantage, work characteristics, and health-related behaviors, may explain SES-related employment gradients observed for depressive symptoms [8]. Likewise, a national study of elderly Chinese adults showed that social participation, via group exercise, mediated 20% of the association between lower income and worse mental health [9]. These findings support KA Matthews and LC Gallo [10]’s proposition that stress and lack of social support in part may explain why low SES and poor health are related. Research has since demonstrated that income inequality (i.e., uneven income distribution across a population) moderates the negative association between relative income (i.e., an individual’s income compared to others) and life satisfaction [11]. This implies that low SES individuals may have worse psychological well-being due to social comparison of income, which evokes feelings of unfairness and lack of trust [12].

Another possible explanation for socioeconomic inequalities in psychological well-being is stigma, defined by E Goffman [13] as the “deeply discrediting attributes” possessed by an individual which exclude them from full social acceptance (pp. 3–4). Stigma is linked to decreased psychological well-being through a convergence of labelling, stereotyping, separation, status loss and discrimination, which occur within power imbalanced contexts [14]. Experiencing stigma or discrimination has been identified as a source of psychological distress in people from lower SES backgrounds, positing SES-based discrimination as a potential mechanism for explaining SES-based inequalities in psychological well-being [15]. A longitudinal cohort study from the Midlife in the United States study (MIDUS) revealed that between 1990 and 2010, the prevalence of, and gaps in daily discrimination received between the highest and lowest SES groups both increased [16]. Using the same dataset, perceived inequality in work and everyday discrimination explained 22% of longitudinal associations between socioeconomic disadvantage and self-rated health [17]. A study from the US showed that amongst women in poverty, internalised stigma (an individual’s internal negative feelings about poverty), and experienced stigma (an individual’s perception of being stigmatized by others) were significant and distinct factors associated with depression [18].

However, there is a dearth of evidence quantifying the extent to which discrimination, specifically attributed to lack of financial resources, explains socioeconomic inequalities in psychological well-being. Recently, A Amirova, KA Rimes and RA Hackett [19] assessed the prevalence of perceived financial-related discrimination in UK and US adults. Perceived financial-related discrimination was defined as any individual who reported experiencing at least one of five discriminative situations (e.g., being threatened or harassed) and then attributed this to their financial status when asked the reason(s) why they believe they were discriminated against. Participants of lower SES were more likely to report experiencing financial-related discrimination compared to higher SES participants, although the study did not assess to the extent to which financial-related discrimination was associated with psychological well-being or explained socioeconomic inequalities in psychological well-being [19].

Compared to generalised or overt stigma and discrimination (e.g., race, weight), financial-related discrimination may be most relevant for understanding socioeconomic inequalities in psychological well-being, given its likely close relation to SES. Therefore, this study aimed to examine if financial-related discrimination increased prospective risk of poorer psychological well-being, and in part explained the development of socioeconomic inequalities across a range of psychological well-being measures. We focused on English older adults from the English Longitudinal Study of Ageing (ELSA), as the only UK longitudinal study to date which has collected information on financial-related discrimination. ELSA also has a diverse range of psychological well-being measures. We hypothesised that perceived financial-related discrimination would be associated with poorer psychological well-being over time, and that it would partly mediate, or explain, prospective associations between wealth (as a measure of SES) and psychological well-being related measures.

## Methods

### Participants

ELSA is an ongoing prospective cohort study initiated in 2002, comprised of 12,099 adults aged ≥ 50 from households who completed the Health Survey for England (HSE) between 1998 and 2001. ELSA participants are interviewed every two years to measure changes in health, social and economic circumstances [20]. This study used data from waves 5 (2010/2011), 6 (2012/2013), and 7 (2014/2015) [21]. Wave 5 was selected as baseline because a range of psychological well-being measures were collected, and this is the only wave to date which measured perceived discrimination, including financial-related discrimination [20]. Using baseline wealth as a SES measure, development of socioeconomic inequalities in a diverse range of psychological well-being measures at Waves 6 (2-year follow-up) and 7 (4-year follow-up) were assessed, to determine the consistency of associations. A total of 10,274 participants contributed data to Wave 5, 9,090 of these being ‘core’ participants who met age eligibility criteria. For the analysis, we included sample weights at baseline (Wave 5) to adjust for non-response. Therefore, we excluded participants without sample weights, resulting in the maximum analytical sample size of 8,988. ELSA has received ethical approval from different institutional review boards for its waves (https://www.elsa-project.ac.uk/ethical-approval). Informed consent was sought from all the ELSA participants.

### Independent variable: Wealth

We used wealth at baseline (Wave 5) as a SES measure in this study. Wealth in ELSA was determined by the total non-pension wealth, including financial wealth from savings and investments, value of home, property, business, and physical wealth assets (e.g., artwork) owned by the respondent/responding couple and dependants, minus any debt [22, 23]. We transformed total non-pension wealth calculated and available in ELSA into tertiles (poorest, middle, richest) (e.g., as in [22]). Table S1 presents distribution of total wealth by tertile. The top tertile (richest) had the biggest range (minimum– maximum values) compared to the lowest (poorest) and middle tertiles.

Even though other SES measures were available (e.g., education, employment status), we examined wealth only, because it was found to be the most robust SES indicator in ELSA, and has the strongest association with mortality in older age [22, 24]. When studying older adults who may be retired or unemployed and not receive income anymore, employment status and income level tend to be less relevant. Therefore, wealth may serve as a better measure, as it captures financial and other resources at older ages more accurately than other traditional SES measures (e.g., income) [23, 25].

### Candidate mediator: Financial-related discrimination

During Wave 5, participants completed an adapted version of the Perceived Everyday Experiences With Discrimination Scale [26], containing five items assessing the frequency and context, of five forms of discriminative treatment: “In your day-to-day life, how often have any of the following things happened to you (1) you are treated with less respect or courtesy; (2) you receive poorer service than other people in restaurants and shops; (3) people act as if they think you are not clever; (4) you are threatened or harassed; (5) you receive poorer service or treatment than other people from doctors or hospitals”. Participants then rated each item on a 7-point scale (1= “almost every day”, 7= “never”). Participants who responded, “almost every day”, “at least once a week”, “a few times a year” or “less than once a year” to any of the five forms of discriminative treatment were then asked to select the reason(s) why they were discriminated against, including “financial status”. Consistent with previous literature examining the association between perceived discrimination and health outcomes [19, 27], financial-related discrimination was defined as participants who reported that their “financial status” was the reason why they were discriminated against. In the current study, whether a participant had experienced financial-related discrimination or not, based on the above criteria, was then dichotomised into “yes” or “no” groups.

### Dependent variables: psychological well-being related measures

Five different psychological well-being related measures at Waves 6 and 7: depressive symptoms, enjoyment of life, eudemonic well-being, life satisfaction and loneliness, were examined, to provide a holistic overview of how psychological well-being related measures may vary according to wealth and perceived financial-related discrimination.

Depressive symptoms were measured using the short-form Center for Epidemiological Studies Depression Scale (CES-D) [28], containing eight items assessing participants feelings over the last week, to which participants responded “yes” or “no”. Negative items (e.g., “You feel depressed?”) were coded as: 0= “no” and 1= “yes”, whereas positively worded items (e.g., “You were happy?”) were reverse coded: 0= “yes” and 1= “no”. This produced a total sum score ranging from 0 to 8, with higher scores indicating greater depressive symptoms (e.g., as in [29]).

Enjoyment of life was measured using four items (e.g., “I enjoy the things that I do,” “I enjoy being in the company of others”), from the Control, Autonomy, Self-Realization and Pleasure (CASP-19) Quality of Life instrument [30]. Participants responded to items on a 4-point scale (0= “never”, 3= “often”), with higher total scores, ranging from 0 to 12, indicating greater enjoyment of life (e.g., as in [31]).

Eudemonic well-being was assessed using the remaining 15 items (e.g., “I feel free to plan for the future”) from the CASP-19 instrument [30]. Similar to the scoring of enjoyment of life, participants responded to each item on a 4-point Likert scale (0= “never”, 3= “often”), with responses to negative items (e.g., “I feel that what happens to me is out of my control”) reverse coded. A total score ranging from 0 to 45 was generated by adding all the items together, with higher scores indicating greater eudemonic well-being (e.g., as in [31]).

Life satisfaction was measured using the Satisfaction with Life scale [32], which contained five items (e.g., “In most ways my life is close to ideal”), which participants responded to on a 7-point Likert scale (1= “strongly agree”, 7= “strongly disagree”). Higher total scores, ranging from 1 to 35, indicated increased life satisfaction (e.g., as in [33]).

Loneliness was quantified using the University of California Los Angeles (ULCA)3-item loneliness scale [34], where participants responded to items (e.g., “How often do you feel you lack companionship?”) with 1= “hardly ever or never”, 2= “some of the time” or 3= “often”. Higher total scores, ranging from 1 to 9, indicated greater loneliness (e.g., as in [35]).

### Covariates

Age (in years), sex (female; male), ethnicity (White; non-White), marital status (single/never married; married; divorced/separated/widowed), education level (degree or equivalent; non-degree) (e.g., as in [31]), employment status (employed; self-employed; retired/semi-retired; unemployed/other) (e.g., as in [36]) and limiting illness status (yes; no), were selected as covariates, as these have been shown to influence psychological well-being and/or perceived discrimination [17, 37].

### Data analysis

#### Primary analyses

Data analysis was conducted using STATA. We included sample weights available at Wave 5 in the analyses to take into account differences in baseline characteristics associated with non-response. Descriptive statistics for categorical variables (sex, ethnicity, marital status, education, employment status, wealth, limiting illness status and financial-related discrimination) were expressed as frequencies and weighted percentages, whereas continuous variables (age) and psychological well-being related measures were expressed as weighted mean and standard deviation (SD). For regression and mediation analyses, psychological well-being related measures across waves were transformed into z-scores (mean = 0; SD = 1), to enable comparison across different matrices.

Cross-sectional associations between wealth and financial-related discrimination at baseline (wave 5) were tested using logistic regression, controlling for baseline sociodemographic covariates. Results were expressed as odds ratio (OR), 95% confidence intervals (CI), and p-values. Linear regressions were then used to examine longitudinal associations between baseline wealth (Wave 5) and psychological well-being related measures at follow-up (Waves 6 and 7), controlling for baseline psychological well-being and sociodemographic covariates (Wave 5). Next, longitudinal associations between financial-related discrimination at baseline (Wave 5) and each psychological well-being related measure at different follow-ups (Waves 6 and 7) were investigated using linear regression, adjusted for baseline psychological well-being and sociodemographic covariates. Results were reported as regression coefficients (β), 95% CI, and p-values.

**Fig. 1:**
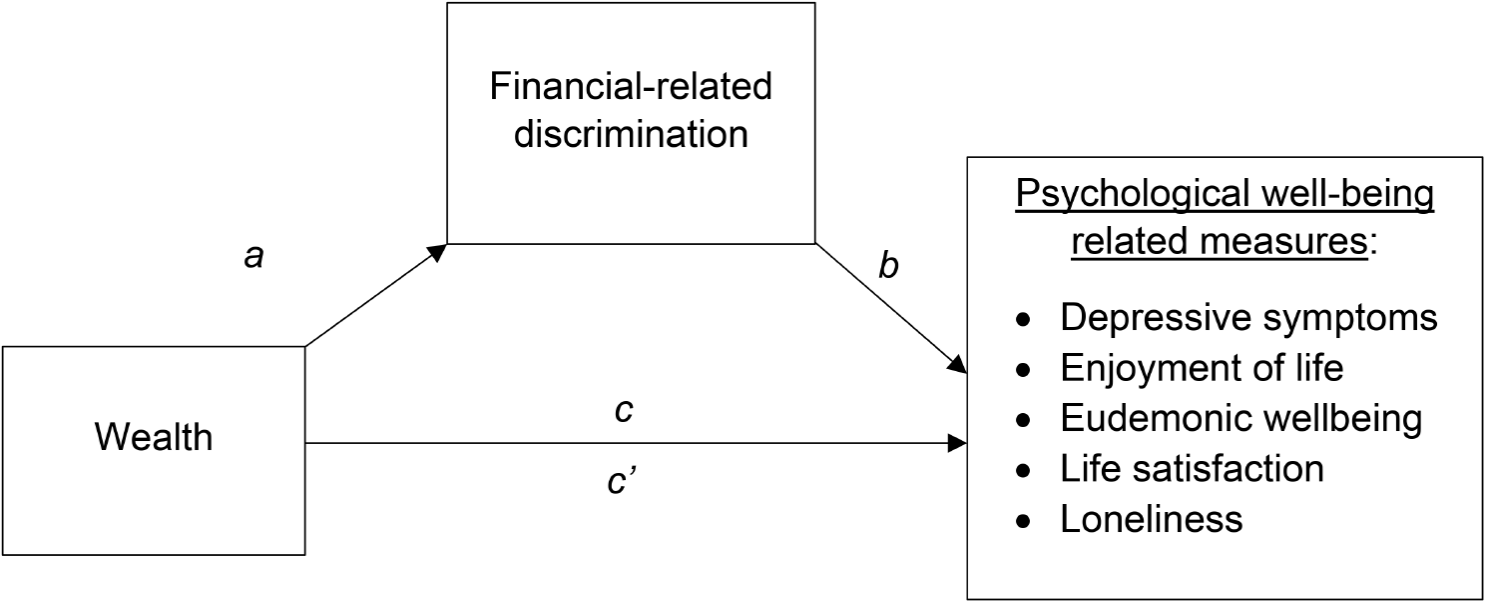
Mediation model. Path a depicts cross-sectional associations between wealth and financial-related discrimination, controlling for baseline psychological well-being related measures and sociodemographic covariates (age, sex, ethnicity, marital status, education, employment status, limiting illness status). Path b illustrates longitudinal associations between financial-related discrimination and psychological well-being related measures at 2-year and 4-year follow-ups, controlling for baseline psychological well-being and sociodemographic covariates. Path c’ represents the direct effect of wealth on psychological well-being related measures longitudinally, in an unmediated model. The indirect effect (path a × b) estimates the longitudinal effect of wealth on psychological well-being related measures via financial-related discrimination. Path c describes the total effect ((a × b) + c’), which estimates the sum of both the indirect and direct effects

Mediation analyses were conducted to examine the role of financial-related discrimination in explaining the association between baseline wealth and follow-up psychological well-being related measures. To ensure mediation preconditions were satisfied [38], the following three associations had to be significant: (1) association between wealth (independent variable– IV) and financial-related discrimination (mediator– M) (path *a*), (2) association between wealth (IV) and psychological well-being (dependent variable– DV) (total effect, path *c*), and (3) association between financial-related discrimination (M) and psychological well-being (DV) (path *b*) (Fig. 1).

Having satisfied mediation preconditions, single mediation models using the Karlson-Holm-Breen (KHB) method [39], were used to examine the mediating role of financial-related discrimination. Within the mediation model, wealth (IV) and financial-related discrimination (M) were both measured at baseline (Wave 5), instead of measuring financial-related discrimination at a separate timepoint after baseline. This was chosen to reduce the possibility that changes in wealth between (baseline– time 1) and immediate wave after baseline (time 2), influence financial-related discrimination, which may then have masked the true effect of baseline wealth on financial-related discrimination. This becomes especially relevant when considering the dynamic nature of wealth variables in older populations, given the unexpected changes to wealth that arise with ageing (e.g., unexpected healthcare costs, costs of managing a long-term health condition) [40]. Our approach for mediation analysis, whereby the independent variable and candidate mediator were measured at the same time point (baseline), was also used by previous studies [31, 41, 42].Mediation findings were presented as total effect (path *c* = (*a* × *b)* + *c’*): combined direct and indirect effect), direct effect (path *c’*), and indirect effect (path *a* × *b*), along with 95% CI and p-values. Effect ratios (indirect effect/total effect), indicating how much the association between wealth index and psychological well-being was explained by financial-related discrimination, were also presented.

As missing values observed in the majority of variables may introduce selection bias, we used multiple imputation by chained equation (MICE) [43, 44] to address missing values in this study. We assumed missing at random as some variables, including age, ethnicity, and marital status, were associated with missingness. We used ‘mi impute chained’ in STATA to create 20 imputed datasets to fill in missing values. We included all variables (independent, mediator, dependent variables) in the imputation model and treated variables with no missing values (age, sex, marital status, see Table 1) as predictors (e.g., as in [45]). Following previous guidelines [46], we also included baseline sample weights to improve the imputation model. Using MICE, the final sample size is the same as the maximum analytical sample size (*n* = 8,988).

**Table 1:** Baseline sociodemographic characteristics, financial related-stigma, and psychological well-being related measures

Variables	n	Unweighted %	Weighted %
Age	8988		
Mean (SD)		67.71 (9.22)	67.26 (9.75)
Sex	8988		
Female	4973	55.33	53.12
Male	4015	44.67	46.88
Ethnicity	8983		
Non white	281	3.13	4.04
White	8702	96.87	95.96
Marital status	8988		
Married/cohabitation	6149	68.41	69.20
Never married/single	465	5.17	5.18
Widowed/divorced/separated	2374	26.41	25.62
Degree level qualification	8910		
No	7442	83.52	85.41
Yes	1468	16.48	14.59
Employment status	8981		
Employed	2009	22.37	24.97
Self employed	531	5.91	6.36
Retired	5395	60.07	56.07
Unemployed/other	1046	16.48	12.60
Limiting illness status	8976		
No	5759	64.16	64.14
Yes	3217	35.84	35.86
Wealth	8843		
Poorest	2951	33.37	35.74
Middle	2947	33.33	32.85
Richest	2945	33.30	31.41
Financial-related discrimination	7937		
No	7463	94.03	93.72
Yes	474	5.97	6.28
Depressive symptoms *(possible range: 0–8)*	8628		
Mean (SD)		1.53 (1.97)	1.58 (2.01)
Enjoyment of life *(possible range: 0–12)*	7992		
Mean (SD)		9.80 (1.86)	9.74 (1.90)
Eudemonic well-being *(possible range: 0–45)*	7976		
Mean (SD)		30.90 (7.50)	30.64 (7.58)
Life satisfaction *(possible range: 1–35)*	7909		
Mean (SD)		25.48 (6.45)	25.36 (6.50)
Loneliness *(possible range: 1–9)*	7968		
Mean (SD)		4.19 (1.55)	4.21 (1.56)

*%=percentage; SD = standard deviation*

*Values were weighted using baseline sample weights at Wave 5*

#### Additional/sensitivity analyses

Findings from previous studies indicated that prior health status may be associated with current material factors (e.g., wealth) [47, 48]. To take into account the influence of earlier psychological well-being on current wealth at baseline, we conducted additional analyses by controlling for pre-baseline psychological well-being at Wave 4 (as opposed to control for baseline psychological well-being at Wave 5) in regression and mediation analyses predicting follow-up psychological well-being (Waves 6, 7).

## Results

### Baseline participant characteristics

Table 1 presents that a roughly equivalent sample of male and female participants were included (47% and 53%, respectively), aged 67.26 years on average. Participants were predominantly White (96%), retired (56%), did not hold a degree level qualification (85%), were married or cohabiting (69%) and did not have a limiting illness (64%). Participants who reported experiencing discrimination attributed to financial-related discrimination was 6%. The proportions of financial-related discrimination were higher amongst participants from the poorest (9%), than those from middle (5%) and richest (4%) wealth groups (Table S2). Participants had favourable psychological well-being at baseline, as the total scores for depressive symptoms and loneliness were lower, and total scores of enjoyment of life, eudemonic well-being, and life satisfaction were higher than 50% of their respective possible total scores (Table 1). This is similar to follow-up psychological well-being (Table S3).

### Associations between wealth and financial-related discrimination

Table 2 depicts adjusted cross-sectional associations between wealth and financial-related discrimination. Participants from the poorest (vs. richest) wealth group were more likely to experience financial-related discrimination (OR = 1.97; 95% CI = 1.49, 2.59). There was no significant difference in the likelihood of experiencing financial-related discrimination between the middle and richest participants (OR = 1.16; 95% CI = 0.86, 1.55). However, compared to the middle group, participants from the poorest group were also more likely to experience financial-related discrimination (OR = 1.70; 95% CI = 1.32, 2.20) (*findings are not presented in* Table 2). For other possible SES measures (e.g., education), we found no strong evidence of the association between these SES measures and financial-related discrimination. Due to larger effect size for the comparison between the poorest vs. richest groups (OR = 1.97) than the poorest vs. middle groups (OR = 1.70), our mediation analysis focused on the poorest vs. richest groups. In addition, the associations between wealth and psychological well-being related measures were presented based on this comparison (the poorest vs. richest).

**Table 2:** Adjusted cross-sectional associations between wealth and financial-related discrimination (*n* = 8,988)

Variables	OR	95% CI
Age	0.96***	0.94, 0.97
Sex (ref: Female)		
Male	1.73***	1.41, 2.12
Ethnicity (ref: White)		
Non white	1.55	0.94, 2.56
Marital status (ref: Married/cohabitation)		
Never married/single	1.01	0.66, 1.55
Widowed/divorced/separated	1.16	0.91, 1.47
Degree Level Qualification (ref: Yes)		
No	1.22	0.89, 1.66
Employment status (ref: Employed)		
Self employed	1.11	0.71, 1.72
Retired	1.18	0.87, 1.61
Unemployed/other	1.41*	1.00, 1.97
Limiting Illness status (ref: No)		
Yes	1.42**	1.14, 1.78
Wealth (ref: Richest)		
Poorest	1.97***	1.49, 2.59
Middle	1.16	0.86, 1.55

ref = reference group; OR = odds ratio; CI = confidence interval

*p < 0.05; **p < 0.01; ***p < 0.001

### Associations between wealth and psychological well-being related measures

Table 3 depicts longitudinal associations between wealth (poorest vs. richest) and psychological well-being related measures at Waves 6 and 7, adjusted for baseline psychological well-being and sociodemographic covariates. Participants from the poorest (vs. richest) group consistently had greater depressive symptoms and lower enjoyment of life, eudemonic wellbeing, and life satisfaction at both 2-year and 4-year follow-up. There were also significant differences in loneliness between the poorest and richest participants for 4-year follow-up only.

**Table 3:** Longitudinal associations between wealth (poorest vs. richest) and psychological well-being related measures (*n* = 8,988)

Variables	Wave 6		Wave 7	
	β	95% CI	β	95% CI
Depressive symptoms	0.18***	0.13, 0.24	0.18***	0.12, 0.24
Enjoyment of Life	-0.14***	-0.19, -0.09	-0.16***	-0.22, -0.11
Eudemonic wellbeing	-0.14***	-0.19, -0.10	-0.16***	-0.21, -0.11
Life satisfaction	-0.09***	-0.14, -0.04	-0.11***	-0.16, -0.06
Loneliness	0.04	-0.01, 0.09	0.06*	0.01, 0.12

β = regression coefficient; CI = confidence intervals

Separate regression models were developed for each psychological well-being related measure, adjusted for baseline psychological well-being and sociodemographic covariates (age, sex, ethnicity, marital status, education level, employment status, and presence of limiting illness)

*p < 0.05; **p < 0.01; ***p < 0.001

### Associations between financial-related discrimination and psychological well-being related measures

Experiencing financial-related discrimination was associated with increased depressive symptoms at 2-year (β = 0.17, 95% CI: 0.07, 0.27) and 4-year follow-ups (β = 0.22, 95% CI: 0.11, 0.33), decreased enjoyment of life at 2-year (β = -0.16, 95% CI: -0.26, -0.07) and 4 year-follow ups (β = -0.12, 95% CI: -0.22, -0.02), and increased loneliness at 2-year (β = 0.12, 95% CI: 0.03, 0.20) and 4-year follow-ups (β = 0.14, 95% CI: 0.04, 0.23) (Table 4). Financial-related discrimination was not associated with eudemonic wellbeing and life satisfaction at both follow-ups. These findings supported our hypothesis that financial-related discrimination may be prospectively associated with worsening of psychological well-being. As per the preconditions for mediation, only depressive symptoms, and enjoyment of life at both follow-ups, and loneliness at 4-year follow-up, were included in subsequent mediation analyses.

**Table 4:** Longitudinal associations between financial-related discrimination (yes vs. no) and psychological well-being related measures (*n* = 8,988)

Variables	Wave 6		Wave 7	
	β	95% CI	β	95% CI
Depressive symptoms	0.17**	0.07, 0.27	0.22***	0.11, 0.33
Enjoyment of Life	-0.16**	-0.26, -0.07	-0.12*	-0.22, -0.02
Eudemonic wellbeing	-0.05	-0.12, 0.03	-0.09	-0.18, 0.00
Life satisfaction	-0.09	-0.18, 0.01	-0.00	-0.10, 0.10
Loneliness	0.12*	0.03, 0.20	0.14**	0.04, 0.23

β = regression coefficient; CI = confidence intervals

Separate regression models were developed for each psychological well-being related measure, adjusted for baseline psychological well-being, wealth, and sociodemographic covariates (age, sex, ethnicity, marital status, education level, employment status, and presence of limiting illness)

*p < 0.05; **p < 0.01; ***p < 0.001

### Mediation by financial-related discrimination

Financial-related discrimination statistically significantly accounted for 3.28% and 4.40% of the total effect of wealth on depressive symptoms at 2-year and 4-year follow-ups, respectively (Table 5). Financial-related discrimination statistically significantly explained 4.17% of the total effect of wealth on decreased enjoyment of life at 2-year follow-up, but did not statistically significantly explain the aforementioned total effect at 4-year follow-up. Financial-related discrimination mediated the association between wealth and loneliness at 4-year follow-up by 7.94%. These results may support the hypothesis that financial-related discrimination may explain the association between wealth and psychological well-being related measures.

**Table 5:** Mediation by financial-related discrimination on the longitudinal associations between wealth (poorest vs. richest) and psychological well-being related measures (*n* = 8,988)

Path	Wave 6			Wave 7		
	Point estimate	95% CI	Effect ratio (%)	Point estimate	95% CI	Effect ratio (%)
**Depressive symptoms**						
Wealth → financial-related discrimination (IV to M, path *a*)	OR = 1.813***	1.373, 2.392		OR = 1.813***	1.373, 2.392	
Financial-related discrimination → depressive symptoms (M to DV, path *b*)	β = 0.173**	0.073, 0.274		β = 0.223***	0.112, 0.334	
Wealth → depressive symptoms (total effect, path *c*)	β = 0.183***	0.128, 0.239		β = 0.182***	0.125, 0.239	
Wealth → depressive symptoms (direct effect, path *c’*)	β = 0.177***	0.122, 0.232		β = 0.174***	0.116, 0.231	
Wealth → depressive symptoms (indirect effect)	β = 0.006*	0.001, 0.011	3.28	β = 0.008*	0.002, 0.014	4.40
**Enjoyment of life**						
Wealth → financial-related discrimination (IV to M, path *a*)	OR = 1.812***	1.371, 2.395		OR = 1.812***	1.371, 2.395	
Financial-related discrimination → enjoyment of life (M to DV, path *b*)	β= -0.160**	-0.255, -0.065		β= -0.116*	-0.215, -0.018	
Wealth → enjoyment of life (total effect, path *c*)	β= -0.144***	-0.193, -0.095		β= -0.164***	-0.221, -0.107	
Wealth → enjoyment of life (direct effect, path *c’*)	β= -0.138***	-0.187, -0.089		β= -0.160***	-0.217, -0.103	
Wealth → enjoyment of life (indirect effect)	β= -0.006*	-0.011, -0.001	4.17	β= -0.004	-0.008, 0.000	2.44
**Loneliness**	NA					
Wealth → financial-related discrimination (IV to M, path *a*)				OR = 1.816***	1.375, 2.398	
Financial-related discrimination → loneliness (M to DV, path *b*)				β = 0.136**	0.037, 0.234	
Wealth → loneliness (total effect, path *c*)				β = 0.063*	0.010, 0.116	
Wealth → loneliness (direct effect, path *c’*)				β = 0.058*	0.005, 0.111	
Wealth → loneliness (indirect effect)				β = 0.005*	0.000, 0.009	7.94

OR = odds ratio; β = regression coefficient; CI = confidence intervals; IV = independent variable; M = mediator; DV = dependent variable; NA = not applicable as the associations between either (1) wealth and psychological well-being or (2) financial-related discrimination and psychological well-being were not statistically significant

The effect ratio was calculated as indirect effect divided by total effect

Separate mediation models were developed for each psychological well-being related measure. All the associations were adjusted for baseline psychological well-being and sociodemographic covariates (age, sex, ethnicity, marital status, education level, employment status, and presence of limiting illness)

*p < 0.05; **p < 0.01; ***p < 0.001

### Findings from additional/sensitivity analyses

Findings from additional analyses controlling for pre-baseline psychological well-being (Wave 4) were consistent for the associations between wealth and follow-up psychological well-being related measures (Table S4). Financial-related discrimination was associated with all psychological well-being related measures at 2-year follow-up, and with depressive symptoms, eudemonic well-being, and loneliness at 4-year follow-up, after controlling pre-baseline psychological well-being (Table S5). Following pre-conditions for mediation, we found consistent evidence that financial-related discrimination mediated the associations between wealth and depressive symptoms at both follow-ups by 3–4% and between wealth and loneliness at 4-year follow-up by 6.85% (Table S6). We found no statistically significant mediation by financial-related discrimination for enjoyment of life and eudemonic well-being. However, mediation by financial-related discrimination was observed for life satisfaction at 2-year follow-up by 3.60%.

## Discussion

The current study examined whether experiencing financial-related discrimination is associated with worsening of psychological well-being related measures, and if it in part explains prospective development of wealth-related socioeconomic based inequalities in psychological well-being related measures amongst older adults. Older adults from the poorest (vs. richest) groups were more likely to report experiencing financial-related discrimination. Financial-related discrimination was prospectively associated with increased depressive symptoms and decreased enjoyment of life at 2-year and 4-year follow-ups, and loneliness at 4-year follow-up, with financial-related discrimination explaining 3–8% of the associations between wealth (poorest vs. richest) and psychological well-being related measures. These findings were largely consistent when we controlled for pre-baseline psychological well-being at Wave 4 in the analyses (as opposed to baseline psychological well-being at Wave 5).

Our findings on the longitudinal associations between wealth and psychological well-being are supported by multiple empirical reports demonstrating clear socioeconomic inequalities in psychological well-being [49, 50]. However, current evidence also indicates that the association between wealth and psychological well-being may not be always positive. A study from the US found that the positive association between income and emotional well-being (i.e., frequency and intensity of emotions which make an individual’s life pleasant or unpleasant) did not progress beyond an income level of $75,000, suggesting that some richer individuals may still experience poor psychological wellbeing [51]. It may be that wealth is attenuated by broader measures of wellbeing amongst older adults such as their levels of resilience, which have been shown to protect older adults against financial difficulties, which may preserve their psychological well-being, despite relatively low wealth [52]. Another study found that extrinsic goal attainment (e.g., pursuit of financial success) positively contributed to despair amongst European older adults, and was unrelated to psychological health, whereas intrinsic goal attainment (e.g., acceptance of death) was associated with higher subjective wellbeing, suggesting the relationship between wealth and wellbeing is more nuanced amongst older populations [53].

To date, the prospective association between financial-related discrimination and psychological well-being related measures has not been examined. The current study furthers knowledge from existing research evidencing the associations between different types of discriminations: weight discrimination [31, 33], sexual-orientation discrimination [54], age discrimination [55] and psychological well-being, by demonstrating how financial-related discrimination may also contribute negatively towards English older adults’ psychological well-being. There are multiple potential mechanisms through which financial-related discrimination may explain longitudinal associations between wealth as a SES measure and psychological well-being. Consistent with IH Meyer [56]’s minority stress framework, which describes relationships between proximal (e.g., perceived discrimination) or distal (e.g., experienced discrimination) stressors and mental distress, perceived financial-related discrimination (as a proximal stressor), may increase the risk of reduced well-being, through stress. This was illustrated by NE Adler and AC Snibbe [57], who produced a model depicting pathways between SES and health, whereby declining SES exposes an individual to increased stress (e.g., financial-related discrimination) and decreased resources to deal with stress (e.g., mental health service availability), resulting in larger psychological responses and increased vulnerability to disease [58]. ML Hatzenbuehler [59] extended the minority stress framework by proposing cognitive (e.g., rumination), affective (e.g., emotion dysregulation) and social (e.g., lack of support) psychological mediators through which proximal or distal minority stressors become associated with psychopathology, which may provide causal mechanisms underlying associations between financial-related discrimination and decreased psychological well-being identified within the current study. This implication is supported by a cross-sectional analysis of US low-income women which revealed internalised poverty stigma and depression were partially mediated by psychological factors like self-esteem and fear of rejection [18].

However, financial-related discrimination only explained a small proportion of prospective associations between wealth and psychological well-being. Significant associations between wealth and psychological well-being, as well as between financial-related discrimination and psychological well-being (independent of wealth), suggest that unmeasured mediators may contribute to this association. For example, a previous study using data of older adults across European countries found that subjective social status (3%) explained the association between SES and life satisfaction [60]. Social support, stress and health-related behaviors may also explain the association between SES and psychological well-being [8, 10]. Furthermore, a relatively low prevalence of financial-related discrimination (6%) in the sample may limit the variability to detect significant findings. In addition, compared to overt racism or sexism, financial-related discrimination may be more difficult to accurately identify or perceive as being caused by financial circumstance, which may make estimating financial-related discrimination prevalence challenging [61]. This may explain why financial-related discrimination was a small yet significant mediator of associations between SES and psychological well-being in the current study.

The relatively small amount of variance that experiencing financial-related discrimination explained for the relationship between wealth and worsening of psychological well-being measures may also be due to the older age of participants in this study. Financial related discrimination was measured at a single time point in old age, and by old age, significant wealth-based inequalities in psychological well-being have already developed. Financial related discrimination experienced earlier in life may therefore play a larger role in the development of socio-economic inequalities in psychological well-being. Although more common among participants in the poorest wealth category (9%), participants in the richest wealth category also reported financial-related discrimination (4%). This suggests that experiencing financial-related discrimination is not limited to very low-income families and may be contributing negatively to the psychological well-being of individuals across socioeconomic statuses.

The use of the health stigma and discrimination framework, designed to inform research and interventions tackling intersecting forms of stigma (e.g., race, gender, class), offers both upstream (e.g., anti-stigma education) and downstream (e.g., targeted psychological therapy) intervention targets [62]. Based on the present results, policy action should address both determinants of financial-related discrimination (e.g., stereotyping, prejudice), whilst supporting individuals most at risk of financial-related discrimination (e.g., low SES) identified within the current study. This may be the most efficacious approach in tackling wealth-related socioeconomic based inequalities in older adults’ psychological well-being.

### Strengths, limitations, and directions for future research

This study is the first to evidence that financial-related discrimination predicts worsening psychological well-being, as well as partially explaining prospective associations between wealth-related SES and psychological well-being related measures. Strengths include use of a longitudinal design, across a nationally representative sample of older adults, which increases the findings generalisability. Additionally, use of MICE to account for missing data across several variables may reduce selection bias. Findings were also largely consistent when either pre-baseline or baseline psychological well-being was controlled for in the analyses.

The study has limitations, including the predominately White, ethnically homogenous sample of English older adults obtained from ELSA, which prevent generalisation of the psychological implications of financial-related discrimination within ethnically diverse populations, or adults aged 18–49. Therefore, future research should measure financial-related discrimination prevalence across populations aged 18–49. Despite that wealth is a likely reliable SES measure given its objectivity, individuals may have had unequal access to household resources, and this would result in an incomplete picture of available financial resources, or the cumulative effect of deprivation, or privilege, prior to retirement [63, 64]. However, previous research found wealth was the most robust SES measure amongst ELSA datasets, compared to any other SES indicator (e.g., education, employment) [22, 24]. The association between wealth and psychological well-being in the current study was less vulnerable to reverse causation, given analyses were controlled for baseline or pre-baseline psychological well-being. However, as wealth and financial-related discrimination were assessed in the same wave, and both measures were self-reported by the participants, this may introduce same-source bias which may influence the findings to some extent.

The current study provides no information on the source of perceived financial-related discrimination and is based on self-report, which is prone to bias. Further research will benefit from better understanding factors contributing to financial related discrimination. SES related variables may interact to increase the risk of experiencing this form of discrimination. H Tajfel and JC Turner [65] suggest that discrimination is more likely to be experienced if an individual is perceived as a member of an outgroup, which may suggest that individuals with a personal SES incongruent to their neighbourhood-level SES may also be more likely to experience financial-related discrimination. Therefore, future research should determine whether neighbourhood-level SES (e.g., index of multiple deprivation) influences individuals’ risk of perceived financial-related discrimination, enabling targeted mental health service provision for individuals most at risk of perceived financial-related discrimination, and subsequently poorer psychological well-being.

Due to information on the experience of discrimination being limited to Wave 5, we were unable to conduct further analyses exploring whether changes in discrimination experiences may moderate the association between wealth and psychological well-being, or the changes in both. Future research may benefit from investigating whether psychological well-being may improve or decline in response to changes in wealth, and whether the related improvement or decline in psychological well-being is more pronounced in those who start experiencing financial-related discrimination, or cease to experience it. When data permit, adopting structural equation models, as demonstrated in [47], can help to examine how changes in psychological well-being, following changes in wealth, may vary based on changes in financial-related discrimination.

## Conclusions

Financial-related discrimination may increase risk for worse psychological well—being and explain a small proportion of socioeconomic inequalities in psychological well-being.

### Electronic supplementary material

Below is the link to the electronic supplementary material.

Supplementary Material 1

## Data Availability

Data are available at https://doi.org/10.5255/UKDA-Series-200011 with the permission of UK Data Services. The codes used to generate the findings presented in this study are available at https://doi.org/10.17605/OSF.IO/2A4MD.
