# Supplementary material for: Financial-related discrimination and socioeconomic inequalities in psychological well-being related measures: a longitudinal study
Source: BMC Public Health. 2024 Apr 11;24:1008. doi: 10.1186/s12889-024-18417-w (PMC11010292; doi:10.1186/s12889-024-18417-w)
Supplement: Supplementary file 1 — Supplementary Material 1 [file 12889_2024_18417_MOESM1_ESM.pdf]

Supplementary materials

Table S1. Distribution of total wealth by tertiles at baseline (Wave 5)

Wealth index	n	Min	Max	Max - Min	Unweighted Mean (SD)	Weighted Mean (SD)
Poorest	2951	-436,410	160,000	596,410	59,434.49 (60,521.45)	55,969.27 (59,640.65)
Middle	2947	160,001	325,100	165,099	233,367.50 (46,488.97)	233.475.20 (46,444.06)
Richest	2945	325,280	10,598,000	10,272,720	707,605.00 (672,915.10)	709,998.10 (688,866.50)

n=number of participants; Min = minimum value, Max=maximum value; SD = standard deviation

Values were weighted using baseline sample weights at Wave 5

Table S2. Distribution of experiencing financial-related discrimination by wealth tertiles at baseline (Wave 5)

**Wealth index**	**Experiencing financial-related discrimination (yes)**	
	**Unweighted %**	**Weighted %**
Poorest	9.30	9.33
Middle	4.87	5.18
Richest	4.12	4.39

%=percentage

Values were weighted using baseline sample weights at Wave 5

Table S3. Distribution of follow-up psychological well-being related measures (Waves 6, 7)

Psychological well-being related measures	n	Unweighted Mean (SD)	Weighted Mean (SD)
**Wave 6**			
Depressive symptoms *(possible range: 0-8)*	7638	1.34 (1.88)	1.39 (1.93)
Enjoyment of life *(possible range: 0-12)*	6980	9.76 (1.89)	9.72 (1.92)
Eudemonic well-being *(possible range: 0-45)*	6951	30.69 (7.50)	30.48 (7.57)
Life satisfaction *(possible range: 1-35)*	6878	25.17 (6.50)	25.06 (6.53)
Loneliness *(possible range: 1-9)*	6940	4.18 (1.54)	4.20 (1.57)
**Wave 7**			
Depressive symptoms *(possible range: 0-8)*	6655	1.35 (1.83)	1.38 (1.86)
Enjoyment of life *(possible range: 0-12)*	6026	9.88 (1.86)	9.84 (1.89)
Eudemonic well-being *(possible range: 0-45)*	6006	31.48 (7.46)	31.30 (7.57)
Life satisfaction *(possible range: 1-35)*	5918	25.76 (6.27)	25.71 (6.33)
Loneliness *(possible range: 1-9)*	5990	4.03 (1.44)	4.04 (1.46)

n=number of participants; SD = standard deviation

Values were weighted using baseline sample weights at Wave 5

Table S4. Longitudinal associations between wealth (poorest vs. richest) and psychological well-being related measures, adjusting for pre-baseline psychological well-being related measures at Wave 4 (n= 8,988)

**Variables**	**Wave 6**		**Wave 7**	
	**β**	**95% CI**	**β**	**95% CI**
Depressive symptoms	0.18***	0.13, 0.23	0.18***	0.12, 0.23
Enjoyment of Life	-0.15***	-0.21, -0.10	-0.18***	-0.24, -0.12
Eudemonic wellbeing	-0.18***	-0.23, -0.13	-0.19***	-0.25, -0.13
Life satisfaction	-0.14***	-0.19, -0.08	-0.15***	-0.22, -0.08
Loneliness	0.06	-0.00, 0.11	0.07*	0.02, 0.13

β = regression coefficient; CI= confidence intervals

Separate regression models were developed for each psychological well-being related measure, adjusted for **pre-baseline psychological well-being** and sociodemographic covariates (age, sex, ethnicity, marital status, education level, employment status, and presence of limiting illness).

*p<0.05; **p<0.01; ***p<0.001

Table S5. Longitudinal associations between financial-related discrimination (yes vs. no) and psychological well-being related measures, adjusted for pre-baseline psychological well-being related measures at Wave 4 (n= 8,988)

**Variables**	**Wave 6**		**Wave 7**	
	**β**	**95% CI**	**β**	**95% CI**
Depressive symptoms	0.17**	0.07, 0.27	0.19***	0.09, 0.30
Enjoyment of Life	-0.11*	-0.21, -0.01	-0.06	-0.17, 0.04
Eudemonic wellbeing	-0.10*	-0.17, -0.02	-0.10*	-0.20, -0.01
Life satisfaction	-0.14**	-0.24, -0.05	-0.03	-0.13, 0.08
Loneliness	0.15**	0.05, 0.24	0.15*	0.03, 0.26

β = regression coefficient; CI= confidence intervals

Separate regression models were developed for each psychological well-being related measure, adjusted for **pre-baseline psychological well-being**, wealth, and sociodemographic covariates (age, sex, ethnicity, marital status, education level, employment status, and presence of limiting illness).

*p<0.05; **p<0.01; ***p<0.001

Table S6. Mediation by financial-related discrimination on the longitudinal associations between wealth (poorest vs. richest) and psychological well-being related measures, adjusted for pre-baseline psychological well-being related measures at Wave 4 (n= 8,988)

Path	Wave 6			Wave 7		
	Point estimate	95% CI	Effect ratio (%)	Point estimate	95% CI	Effect ratio (%)
**Depressive symptoms**						
Wealth → financial-related discrimination (IV to M, path *a*)	OR= 1.764***	1.315, 2.365		OR= 1.764***	1.315, 2.365	
Financial-related discrimination → depressive symptoms (M to DV, path *b*)	β= 0.167**	0.065, 0.268		β= 0.194***	0.088, 0.301	
Wealth → depressive symptoms (total effect, path *c*)	β= 0.182***	0.130, 0.234		β= 0.175***	0.115, 0.235	
Wealth → depressive symptoms (direct effect, path *c′*)	β= 0.176***	0.124, 0.229		β= 0.168***	0.108, 0.228	
Wealth → depressive symptoms (indirect effect)	β= 0.006*	0.001, 0.011	3.30	β= 0.007*	0.001, 0.012	4.00
**Enjoyment of life**				NA		
Wealth → financial-related discrimination (IV to M, path *a*)	OR= 1.721***	1.285, 2.305				
Financial-related discrimination → enjoyment of life (M to DV, path *b*)	β= -0.108*	-0.206, -0.009				
Wealth → enjoyment of life (total effect, path *c*)	β= -0.151***	-0.205, -0.097				
Wealth → enjoyment of life (direct effect, path *c′*)	β= -0.147***	-0.202, -0.093				
Wealth → enjoyment of life (indirect effect)	β= -0.004	-0.008, 0.001	2.65			

Path	Wave 6			Wave 7		
	Point estimate	95% CI	Effect ratio (%)	Point estimate	95% CI	Effect ratio (%)
**Eudemonic well-being**						
Wealth → financial-related discrimination (IV to M, path *a*)	OR= 1.444*	1.063, 1.962		OR= 1.444*	1.063, 1.962	
Financial-related discrimination → eudemonic well-being (M to DV, path *b*)	β= -0.096*	-0.174, -0.017		β= -0.102*	-0.195, -0.010	
Wealth → eudemonic well-being (total effect, path *c*)	β= -0.179***	-0.231, -0.126		β= -0.189***	-0.247, -0.130	
Wealth → eudemonic well-being (direct effect, path *c′*)	β= -0.176***	-0.229, -0.124		β= -0.186***	-0.245, -0.128	
Wealth → eudemonic well-being (indirect effect)	β= -0.002	-0.005, -0.001	1.12	β= -0.002	-0.006, 0.001	1.06
**Life satisfaction**				NA		
Wealth → financial-related discrimination (IV to M, path *a*)	OR= 1.738***	1.298, 2.326				
Financial-related discrimination → life satisfaction (M to DV, path *b*)	β= -0.142**	-0.238, -0.047				
Wealth → life satisfaction (total effect, path *c*)	β= -0.139***	-0.194, -0.084				
Wealth → life satisfaction (direct effect, path *c′*)	β= -0.134***	-0.189, -0.079				
Wealth → life satisfaction (indirect effect)	β= -0.005*	-0.010, -0.000	3.60			

Path	Wave 6			Wave 7		
	Point estimate	95% CI	Effect ratio (%)	Point estimate	95% CI	Effect ratio (%)
**Loneliness**	NA					
Wealth → financial-related discrimination (IV to M, path *a*)				OR= 1.798***	1.343, 2.406	
Financial-related discrimination → loneliness (M to DV, path *b*)				β= 0.145*	0.031, 0.258	
Wealth → loneliness (total effect, path *c*)				β= 0.073*	0.016, 0.131	
Wealth → loneliness (direct effect, path *c′*)				β= 0.068*	0.010, 0.126	
Wealth → loneliness (indirect effect)				β= 0.005*	0.000, 0.010	6.85

OR= odds ratio; β = regression coefficient; CI= confidence intervals; IV= independent variable; M= mediator; DV= dependent variable; NA = not applicable as the associations between either 1) wealth and psychological well-being or 2) financial-related discrimination and psychological well-being were not statistically significant.

The effect ratio was calculated as indirect effect divided by total effect.

Separate mediation models were developed for each psychological well-being related measure. All the associations were adjusted for **pre-baseline psychological well-being** and sociodemographic covariates (age, sex, ethnicity, marital status, education level, employment status, and presence of limiting illness).

*p<0.05; **p<0.01; ***p<0.001
